# Can Tumor Necrosis Factor-****α**** and Interleukin-6 Be Used as Prognostic Markers of Infection following Ureteroscopic Lithotripsy?

**DOI:** 10.1155/2014/457063

**Published:** 2014-01-30

**Authors:** Athanasios Bantis, Georgios Tsakaldimis, Athanasios Zissimopoulos, Stilianos Giannakopoulos, Christos Kalaitzis, Michail Pitiakoudis, Alexandros Polichronidis, Stavros Touloupidis

**Affiliations:** ^1^Urology Department, University Hospital of Alexandroupolis, 68100 Thrace, Greece; ^2^Nuclear Medicine Department, University Hospital of Alexandroupolis, 68100 Thrace, Greece; ^3^General Surgery Department, University Hospital of Alexandroupolis, 68100 Thrace, Greece

## Abstract

*Introduction*. Ureteroscopic lithotripsy (URS) although highly effective for the treatment of ureteral stones is associated with certain complications, the more common of which are postoperative fever and infection. In the present study we aimed to evaluate the levels of serum cytokines in patients undergoing ureteroscopic lithotripsy and investigate any possible correlation between levels of cytokines and infectious complications after URS. *Materials and Methods*. Thirty patients (19 males, 11 females), with a mean age of 47 (range: 26–68) that underwent URS lithotripsy for ureteral stones, and 10 healthy volunteers serving as the control group were enrolled in this study. Serum samples for TNF-**α** and IL-6 were obtained before surgical intervention and after 1, 24, and 48 hours and 2 , 24, and 48 hours, respectively. The preoperative and postoperative levels were compared and correlated with the possible complications after URS. *Results*. Serum TNF-**α** levels were statistically significant, increased 1 hour (*P* = 0.0083) and 48 hours (*P* < 0.001) after operation. IL-6 levels were found statistically significant, elevated after 2 and 24 hours from the URS (*P* < 0.001). In 2 patients we observed postoperative fever (>38.5°C). These two patients had high preoperative values of TNF-**α** and IL-6 ( 30 and 50 pg/mL, resp.) and these values increased postoperatively. *Conclusion*. High preoperative levels of serum TNF-**α** and IL-6 may indicate a predisposition for postoperative inflammation and infection following URS lithotripsy.

## 1. Introduction

Urolithiasis is one of the most common urological diseases with various surgical treatments available for its management. One of most utilized surgical modalities is ureteroscopy (URS) with endoscopic stone lithotripsy [1]. URS, either rigid or flexible, is the treatment of choice for almost all ureteral stones and for certain cases of intrarenal lithiasis. Although its therapeutic benefits are recognized, URS is associated with complications that range from pain and hematuria to ureteral perforation, ureteral avulsion, fever, sepsis, and death [2].

With regard to the infectious complications of URS lithotripsy, there are certain serum markers including pro-inflammatory cytokines that are gaining importance in clinical practice [3–5]. Cytokines are a group of peptides that regulate the humoral and cellular components of the immune system and *in vivo* inflammatory responses. Interleukin-6 (IL-6) is an inducer of activation and differentiation of B and T cells during inflammatory responses. IL-6 also activates the vascular endothelium in the process of inflammation [6]. Tumor necrosis factor-*α* (TNF-*α*) is produced by many cells *in vivo*. Increased and prolonged release of TNF-*α* is harmful and causes inflammation and tissue damage [7].

In the present study, we used serum levels of IL-6 and TNF-*α* as markers of inflammation in order to investigate the potential association between these markers and the infectious complications of URS lithotripsy.

## 2. Materials and Methods

Thirty patients (19 males, 11 females), mean age of 47 (range: 26–68) with ureteral lithiasis treated with ureteroscopic lithotripsy, and 10 healthy volunteers serving as the control group were studied. The demographic characteristics of both groups are shown in Table 1. None of the patients treated with URS had received previous treatment for stones (URS, PCNL, or ESW) and none had a history of systemic or immunosuppressive disease. Other exclusion criteria included age less than 18 or more than 80, previous insertion of a ureteral stent or nephrostomy tube, neoplasmatic disease, and renal insufficiency. Urine cultures, before and after URS, were routinely obtained in all patients in order to exclude the presence of urinary tract infection. All URS cases were performed at the dedicated Stone Center of the University Hospital of Alexandroupolis. All cases were performed under general anesthesia. A 8.5 F semirigid ureteroscope (Olympus Medical Systems Europa GmbH) was used and lithotripsy was performed using a Holmium Laser lithotripter (Dornier Medilas Med tech EMEA). Standard rigid forceps or disposable baskets (COOK Medical, Bloomington, IN, USA) were selectively used for retrieval of stone fragments. A double-J catheter was usually left after the surgical intervention depending on the surgeon's discretion.

Serum levels of both TNF-*α* and IL-6 were measured in all patients before the procedure (previous day) and at certain time points following the URS. TNF-*α* was measured at 1 hour postoperatively and again at 24 and 48 hours postoperatively, while IL-6 levels were measured at 2 hours postoperatively and again 24 and 48 hours after the case. The samples were centrifuged at 4000 rpm for 10 minutes at 4°. The serum samples were divided into aliquots and stored at −85°C for the assessment performed in weekly intervals. Serum interleukin-6 concentrations were measured using the commercial Biosource Europe SA IL-6- IRMA and TNF-*α*- IRMA (Rue de l'Industrie 8 B-1400 Nivelles, Belgium) kit (code 30 126 00 for IL-6 and 30 175 20 for TNF-*α*) by immunoradiometric assay **(**IRMA**) **methods. The reference range for IL-6 normal values was 6–31 pg/mL for healthy controls and for TNF-*α* 5 pg/mL, respectively.

## 3. Statistical Analysis

Statistical analysis was performed using the statistical package SPSS V.11. Statistical analyses of serum TNF-*α* and IL-6 values of control group and 1 (or 2 for IL-6), 24, and 48 hours after URS were performed using bivariate linear correlation model between the groups of interest (Pearson's test). The dependence of serum TNF-*α* and IL-6 values with the other variables (blood sample collections after URS) was assessed using paired samples *t*-test with confidence interval 95%. A *P* value of less than 0.01 was considered statistically significant.

## 4. Results

We found significant differences between the cytokine levels in the serum samples taken before and two hours after URS (independent variable the control group) (Table 2).

**Figure 1:**
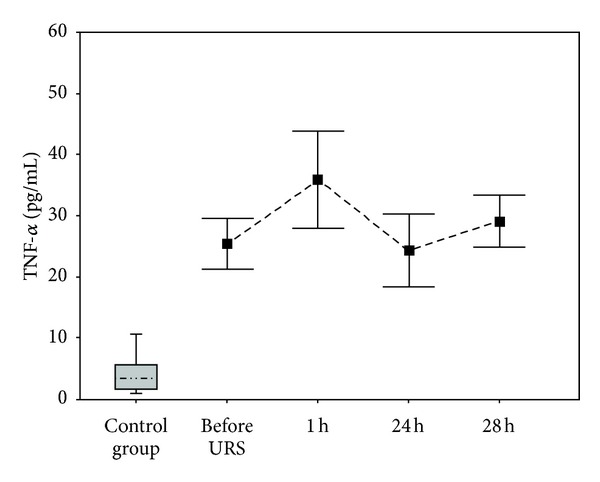
Distribution of serum TNF-*α* levels.

**Figure 2:**
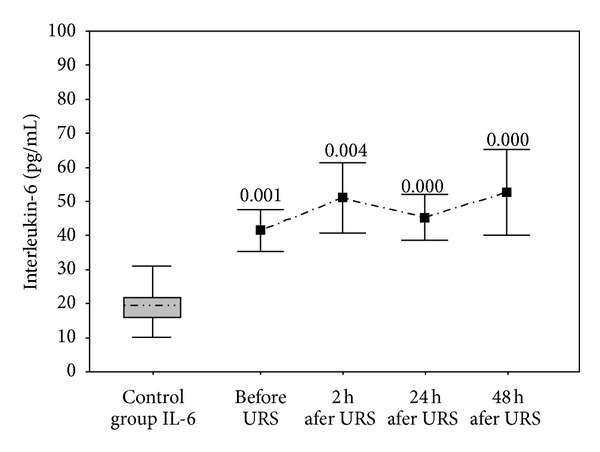
Distribution of serum IL-6 levels.

Correlations and *P* values <0.001 are shown in Figures 1 and 2. Some changes in serum cytokine levels were observed in bivariate linear correlation between the groups of interest (Pearson's test) and are shown in Table 3. Serum TNF-*α* levels were significantly increased after one hour (*P* = 0.0083) and 28 hours (*P* < 0.001) after URS. IL-6 was also significantly increased after 2 and 24 hours of URS (*P* < 0.001). Macroscopic hematuria was observed in 4 patients, while in the remaining 26 patients microscopic hematuria was detected.

Postoperative fever (>38.5°C) was developed in 2 patients (6,6%) and was managed with i.v antibiotics (ciprofloxacin 1 gr/day for 7 days). Both patients had negative urine culture both before and after URS and both had double-J stents inserted at the end of the case. In these two patients the preoperative values for TNF-*α* were 30 pg/mL, 22 pg/mL for TNF-*α*, and 50 and 48 pg/mL for IL6. Postoperatively in one hour for TNF-*α* the serum values increased to 40, 45 pg/mL and in two hours for IL increased to 62, 59 pg/mL.

## 5. Discussion

URS although highly effective in the treatment of ureteral stones may be associated with certain complications [8]. Complications related to inflammation and infection are those more commonly seen after URS lithotripsy. In an effort to predict the patients that are more prone to inflammatory complications following URS a number of potential markers have been proposed [9].

Activation of local and systemic metabolic response *σ* to trauma and systemic inflammatory response syndrome (SIRS) is mediated mainly by activation of interleukins (IL) and tumor necrosis factor-*α* (TNF-*α*) [10].

Once IL and TNF are secreted, they activate several other reactions exacerbating the host inflammatory response. *In vitro* human blood monocytes produce IL and TNF-*α* when they are exposed to 25 to 50 pg/mL of endotoxin concentration. These endotoxin levels have been reported in the bloodstream of patients during septic shock [11].

In a clinical report of 97 consecutive patients, 56% developed sepsis with about 26 pg/mL of TNF-*α* 37% had 20 pg/mL of IL-1 and in 80%; 415 pg/mL of IL-6 was detected, including a LPS mean concentration of 2.6 endotoxin units (EU)/mL (1 EU/mL = 0.6 ng/mL) [12].

In our study we observed that patients with a postoperative fever showed a higher preoperative expression of serum TNF-*α* and IL6. These high levels were maintained and increased in one hour (for TNF-*α* 40 and 45 pg/mL) and two hours (for IL 62 and 59 pg/mL). These two patients were treated with intravenous antibiotics and required more days of hospitalization (4 additional days). Infection and fever were considered as an unpleasant postoperative complication possibly unrelated to the minimally invasive nature of URS.

Serum IL-6 level is a suitable indicator for clinical purposes, because its peak can be measured even on the day after the operation (14 to 20 hours after surgery). In the study by Igarashi et al. serum IL-6 levels differed according to operative procedures. Briefly, open surgery resulted in the most exaggerated increase in serum IL-6 levels, followed by laparoscopic surgery, endoscopy, and ESWL [4].

On the other hand TNF levels increase within 1 hour and then return to baseline within 3 hours after endotoxin administration [13]. Also the plasma concentration of IL-6 showed an increase in 2 to 4 hours following intravenous endotoxin [14]. IL-1 was detected in 60 min and high levels occurred in 3 hours following lipopolysaccharide stimulation of monocytes [15]. Peak levels of IL-1b were also observed at 3 hours during experimental endotoxemia [16]. Based on this data, in our study the cytokine levels were measured at the first and second hour for TNF-*α* and IL-6, respectively, after URS. The significance in the increase of serum TNF-*α* and IL-6 levels may be due to the sampling method, which serum once at a specific time. Serum TNF-*α* levels were statistically significant, increased 1 hour (*P* = 0.0083) and 48 hours (*P* < 0.001) after operation. IL-6 levels were found statistically significant, elevated after 2 and 24 hours from the URS (*P* < 0.001). Furthermore our series revealed that the serum IL-6 and TNF-*α* levels differed according to postoperative timing of blood collection. As indicated in Table 2 the mean value for serum IL-6 before URS was 24 pg/mL and 41,74 36,87 66,54 for 2, 24, and 48 hours after URS and for TNF-*α* the values were 19,90 40,09 17,19 and 26,28 before URS 1, 24, and 48 hours postoperatively.

Limitations of our study include its small size and also the lack of data on stone composition and culture. However we were able to show that cytokines could be helpful as markers of inflammation and potential infectious complications following URS with more accuracy than urine culture. Therefore despite the above mentioned limitations, we consider that our results may be helpful in designing future studies that will address the clinical significance of serum markers of inflammation in the management of patients with stones.

## 6. Conclusions

Serum IL-6 and TNF-*α* levels are affected in ureteroscopic lithotripsy. High preoperative levels of serum TNF-*α* and IL-6 may be indicators of an inflammatory predisposition and could be used as preoperative markers. Further studies that measure the cytokine levels at various intervals before and after URS lithotripsy treatment may help to investigate the possible association between preoperative serum values and postoperatively infectious complications.

## Figures and Tables

**Table 1:** Clinical characteristics of 30 patients with ureter lithiasis treated with Holmium Laser lithotripsy and 10 health donors of control group.

	Patients with stones	Control group
Mean age (years) (range)	47 (26–68)	51,05 (31–72)
Gender ♀/♂	19/11	6/4
Stone size (cm)	Mean stone size	
0–0,6	3 (10%)	—
0,6–1.00	15 (15%)	—
>1.01	12 (12%)	—
Stone location		
Proximal ureter	4 (13.3%)	—
Midureter	13 (43.3%)	—
Distal ureter	13 (43.3%)	—

**Table 2:** Paired samples *t*-test with confidence interval 95%. Independent variable the control group of IL-6 and TNF-*α* before and 1 or 2, 24, and 48 hours after ureteroscopy (statistically significant when *P* value < 0.01).

	Paired differences	Std. deviation	Std. error mean	95% confidence interval of the difference		*t*	df	Sig. (2-tailed)
	Mean			Lower	Upper			
Interleukin-6								
Before URS-CG	24,0490	19,2386	6,0838	10,2865	37,8115	3,953	9	.003
2 h after URS-CG	41,7824	39,7444	12,5683	13,3510	70,2138	3,324	9	.009
24 h after URS-CG	36,8700	19,0497	6,0240	23,2427	50,4973	6,120	9	.000
48 h after URS-CG	66,5490	46,0981	14,5775	33,5724	99,5256	4,565	9	.001
TNF-*α*								
Before URS-CG	19,9040	12,7945	4,0460	10,7514	29,0566	4,919	9	.001
1 h after URS-CG	40,0930	32,4138	10,2501	16,9056	63,2804	3,911	9	.004
24 h after URS-CG	17,1900	9,6078	3,0383	10,3170	24,0630	5,658	9	.000
48 h after URS-CG	26,2840	13,3134	4,2101	16,7602	35,8078	6,243	9	.000

**Table 3:** Bivariate linear correlation between the groups of interest (Pearson's test).

		Before URS	1 h after URS	24 h after URS	48 h after URS
TNF-*α*					
Before URS	PC *P* value *N*				
1 h after URS	PC *P* value *N*	472* .0083 0			
24 h after URS	PC *P* value *N*	.228 225 30	.045 .813 30		
48 h after URS	PC *P* value *N*	.858** .000 30	.565** .001 30	.140 .462 30	
Interleukin-6					
Before URS	PC *P* value *N*				
2 h after URS	PC *P* value *N*	.630** .000 30			
24 h after URS	PC *P* value *N*	.611** .000 30	.483** .007 30		
48 h after URS	PC *P* value *N*	.409* .025 30	.281 .132 30	.676** .000 30	

**Correlation is significant at the 0.01 level (2-tailed).

*Correlation is significant at the 0.05 level (2-tailed).

PC: Pearson correlation.

*P* value: Sig. (2-tailed).
